# The Position of Inhaled Chemotherapy in the Care of Patients with Lung Tumors: Clinical Feasibility and Indications According to Recent Pharmaceutical Progresses

**DOI:** 10.3390/cancers11030329

**Published:** 2019-03-07

**Authors:** Rémi Rosière, Thierry Berghmans, Paul De Vuyst, Karim Amighi, Nathalie Wauthoz

**Affiliations:** 1 Unité de Pharmacie Galénique et de Biopharmacie, Faculté de Pharmacie, Université libre de Bruxelles (ULB), Brussels 1050, Belgium; kamighi@ulb.ac.be (K.A.); nawautho@ulb.ac.be (N.W.); 2 Service des Soins Intensifs et Urgences Oncologiques et Oncologie Thoracique, Institut Jules Bordet, Université libre de Bruxelles (ULB), Brussels 1000, Belgium; thierry.berghmans@bordet.be; 3 Service of Pneumologie, Hôpital Erasme, Université libre de Bruxelles (ULB), Brussels 1070, Belgium; Paul.De.Vuyst@erasme.ulb.ac.be

**Keywords:** pulmonary delivery, lung cancer chemotherapy, non-small cell lung cancer, metastases

## Abstract

Despite new treatment modalities, including targeted therapies and checkpoint inhibitors, cytotoxic chemotherapy remains central in the care of patients with lung tumors. Use of the pulmonary route to deliver chemotherapy has been proved to be feasible and safe in phase I, Ib/IIa and II trials for lung tumors, with the administration of drug doses to the lungs without prior distribution in the organism. The severe systemic toxicities commonly observed with conventional systemic chemotherapy are consequently reduced. However, development has failed in phase II at best. This review first focuses on the causes of failure of inhaled chemotherapy. It then presents new promising technologies able to take up the current challenges. These technologies include the use of a dry powder inhaler or a smart nebulizer with advanced drug formulations such as controlled-release formulations and nanomedicine. Finally, the potential position of inhaled chemotherapy in patient care is discussed and some indications are proposed based on the literature.

## 1. Introduction

Chemotherapy remains the backbone of the care of patients with lung tumors, including advanced primary tumors and lung metastases. This is despite severe systemic toxicities that are due to chemotherapy’s poor selectivity for tumor cells compared to normal cells and the use of systemic routes of administration, i.e., mainly the intravenous (iv) route, which results in a distribution of the drug to the entire organism. To decrease these toxicities, the use of the pulmonary route is promising. Pulmonary drug delivery is well-established for treating many respiratory diseases, such as asthma and chronic obstructive pulmonary disease (COPD) [1]. Compared with systemic delivery (i.e., through enteral and parenteral routes), pulmonary delivery allows high local drug concentrations and low systemic exposure, i.e., it increases the therapeutic ratio. In theory, this approach should therefore be highly beneficial in the care of patients with lung tumors.

The first clinical report on inhaled chemotherapy was published in 1968 [2]. Since then, many clinical trials have been conducted in different populations of patients with lung cancer [3,4,5,6,7,8,9] or lung metastases [5,10,11]. Although inhaled chemotherapy has been proved to be feasible and safe in most of these trials, all the products involved have failed in phase II at best [9]. This narrative review first focuses on the main causes of failure of inhaled chemotherapy in clinical trials. Key clinical reports are discussed. Then, alternative approaches and new technologies that are undergoing preclinical research are reviewed. Finally, the possible position of inhaled chemotherapy in patient care is discussed and potential indications are suggested. It must be noted that the present review only focuses on inhaled chemotherapy. It does not include inhaled therapies such as inhaled gene therapy or immunotherapy, although these are also promising.

## 2. Inhaled Chemotherapy in Clinical Trials: What Are the Causes of Failure?

In view of the clear advantages of administering a local treatment, delivering chemotherapy directly to the lungs to treat lung tumors has been considered for several decades. As nebulization is the simplest method to deliver a drug to the lungs, inhalation has been achieved by nebulizing liquid drug formulations, i.e., iv solution-like formulations, in the first studies, and liposomal dispersions. The state of the art in clinical trials has been reviewed in different works [12,13,14,15].

### 2.1. An Irrational Approach Regarding Lung Deposition, Tumor Penetration and Toxicity

Pulmonary drug delivery has been long considered as an irrational option for administering chemotherapy. It seems to be difficult for pulmonologists to accept that an inhaled treatment could be effective on large solid lung tumors. The part of tumor accessible through the airways would only be the ‘top of the iceberg’, i.e., the extremities of the lesions in direct contact with inhaled air.

Moreover, it seems logical to consider that the largest part of the inhaled dose will be deposited in the healthy adjacent lobes and/or contralateral lung. As 10–30% of chemotherapies in lung cancer therapy may induce lung toxicities [16], it was thought that concentrating these compounds in the respiratory tract might dramatically increase the severity of these toxicities. Selection of the best chemotherapeutic candidate to investigate the pulmonary route is therefore crucial. For all the drug candidates selected in clinical trials (i.e., cisplatin [3,7,11], carboplatin [9], gemcitabine [6], doxorubicin [8,10], 9-nitrocamptothecin [5], 5-FU [4]), inhalation was considered as safe by the investigators. However, the most severe toxicities observed in these trials were related to the pulmonary tract, including the dose-limiting toxicity (DLT) in phase I (for gemcitabine, doxorubicin, 9-NC and 5-FU). It should be noted that vesicant drugs such as doxorubicin generated more severe pulmonary toxicities than other drugs such as 9-NC. Most of the chemotherapy-related conventional systemic toxicities were not observed. Moreover, as lung cancer patients often have impaired pulmonary function consecutive to tobacco use and/or subjacent lung diseases such as COPD [6,17], they can therefore be more subject to severe local adverse effects related to inhaled chemotherapy [6]. Pulmonary toxicity remains one of the main challenges in inhaled chemotherapy and its intensity is directly related to the drug and its dose [6,12,18,19].

Additionally, drug deposition in tumor-bearing lungs might also be a concern. Lemarie et al. determined aerosol deposition by scintigraphy using a ^99m^Tc derivative as a tracer of the gemcitabine aerosol [6]. Aerosol deposition was strongly correlated with lung ventilation but not with the tumor location.

### 2.2. The Need to Administer High Local Chemotherapy Doses

Another problem is the need to administer high drug doses to lung cancer patients as high doses are non-conventional in inhaled therapy for asthma or COPD [19]. Using nebulization, three main parameters are critical for delivering high drug doses. These parameters include (i) the drug concentration and therefore the drug solubility in the formulation to be nebulized, (ii) the nebulizer performance in terms of rate of nebulization (usually in the range of 0.2–0.3 mL/min) and (iii) the fraction of the drug dose deposited in the lungs (usually in the range of 10–15%) [14,19,20].

Wittgen et al. reported that DLT was not reached in phase I for nebulized liposomal cisplatin [7]. The authors’ explanations were (i) the low cisplatin concentration in the formulation (1 mg/mL), (ii) the poor performance of the nebulizer in terms of lung deposition (10–15% of the nominal dose deposited in the lungs) and (iii) pressure generated inducing a nebulization rate of up to 0.3 mL/min. In addition, cisplatin is a small molecule that is rapidly eliminated from the lungs [3] due to rapid systemic absorption (into the blood and lymphatic circulations), which can diminish the intended therapeutic effect. The maximum delivered dose within a cycle was 60 mg/m² (cumulative dose of 120 mg/m²). This dose was administered using a nebulization duration of 20 min, with a maximum of three consecutive nebulizations per session and a maximum of three sessions per day with a rest of 3 h between each session. This dose was administered for three consecutive days/14-day treatment cycle in two cycles. The highest dose delivered within one week was 80 mg and was administered to one patient [7]. This timing would not be easily feasible in patient care practice. 

This timing limitation is mainly related to the water-solubility of the chemotherapeutic agents, which is the major limitation in nebulization formulations. Many chemotherapeutic compounds are poorly water-soluble or insoluble. This significantly limits drug concentrations in the formulation. These compounds therefore require relatively more complex drug formulations They necessitate the use of excipients or co-solvents to increase drug solubility. As for iv formulations, osmolality of these nebulized formulations must be adjusted to approximately 300 mosmol/L and pH adjusted to neutrality as the lungs have limited buffering capacity [21]. However, in contrast to iv delivery, which leads to a high dilution of the formulation in the systemic circulation, inhalation of small volumes of these complex formulations might be poorly locally tolerated [21,22]. For example, to be nebulized in phases I and I/II, doxorubicin was solubilized in a 20% ethanol aqueous solution at pH 3 [8,10]. This composition might be responsible for local adverse effects (e.g., caught, bronchospasms, dyspnoea, bronchitis). Another example is the conventional surfactant/solvent-based iv formulation of paclitaxel, which includes Cremophor EL and ethanol (1:1 v/v). This formulation cannot be considered for inhalation because of the presumed poor tolerability of its components by the respiratory tract [22,23]. However, because gemcitabine and carboplatin are water-soluble compounds, their nebulized formulations have consisted of simple aqueous solutions [6,9]. In one study, nebulized gemcitabine was administered in doses of up to 4 mg/kg within a nebulization time of up to about 30 min [6]. Here, the dose fraction deposited in the lungs was 42 ± 16% (expressed in terms of dose in the nebulizer). This fraction is higher than is usually observed with other nebulizers (usually 10–15% [7,9]). However, the DLT was a bronchospasm at 4 mg/kg [6].

### 2.3. The Management of Air Contamination by the Aerosol

Inhaled chemotherapy requires particular infrastructure to manage air contamination by the aerosol and ensure the safety of the medical staff. The nebulizers used in clinical trials have been equipped with different devices to limit aerosol losses in the air. These devices have included filters collecting exhaled aerosols (Aero-Tech II), apparatus for mouth-only inhalation (OncoMyst model CDD-2a) and breath-enhanced nebulizers (Pari LC Plus et Pari LC Star) that increase lung deposition and reduce administration time [7,10]. During nebulization, patients have been located in depressurized ‘tents’ or ‘cabins’ equipped with an air extractor and with both activated charcoal and high efficient particulate air (HEPA) filters, which capture at least 99.97% of particles ≥0.3 µm [5,7,9,10,11,24]. A check of the safety of this equipment is recommended. For instance, in the study with cisplatin liposomes [24], investigators demonstrated undetectable cisplatin concentrations in the environment that were below the established limit of exposure to cisplatin, i.e., max 2 ng/L in the air for a maximum of 8 consecutive hours. Moreover, a patient’s exhaled air can contain relatively large drug doses (e.g., 11% of the gemcitabine dose in the nebulizer has been found in exhalation filters [6]). All these safety adaptations, in addition of being not patient-friendly, have a financial cost and limit potential clinical development of inhaled chemotherapy. This cost, in addition to the modest improvements observed, has indubitably played a role in the discontinuation of clinical development to further phases [8].

### 2.4. The Current Lack of Clinical Data on Efficacy

As the most advanced development of inhaled chemotherapy has been conducted up to phase II, there is a lack of clinical data on efficacy. However, although the patients involved in clinical trials presented at an advanced stage (often stage IV with no response to first and second line therapies) in most of the phase Ib/IIa trials, they responded to inhaled chemotherapy with a complete, partial or stable response [15]. In the only phase II conducted so far, a significant increase in survival was observed in patients receiving one-third the carboplatin dose by inhalation in a carboplatin/docetaxel doublet compared to patients receiving the same iv doublet (275 days (95% CI 249–300) vs. 211 (95% CI 185–236), *p* < 0.001) [9].

## 3. Ongoing Preclinical Research

Many recent innovative studies have been conducted in this field and recently reviewed [14,19,20,25,26,27]. Here, we present what we consider to be the most promising technologies that take up the main challenges encountered in inhaled chemotherapy. These challenges are (i) to obtain a safe pulmonary profile, (ii) to administer therapeutic doses within a reasonable time, (iii) to maintain therapeutic drug concentrations into the tumor site for a sufficient time and (iv) to limit environmental contamination by the aerosol during an inhalation session (Table 1). 

### 3.1. Tailored Inhalation Devices: Dry Powder Inhalers (DPIs) and ‘Smart’ Inhalers

Dry powder inhalers (DPIs) as inhalation devices for inhaled chemotherapy are promising [19]. Although nebulizers have been the only type of inhalation device used for inhaled chemotherapy in clinical trials so far [15], DPIs have also been considered [28,29,36]. Compared to nebulizers, DPIs are able to deliver high drug doses within a short time period (less than a minute) (Table 1). Levet et al. developed DPI formulations with up to 75% cisplatin loading [28]. By means of these dry powder formulations, a deposited cisplatin dose of 9 mg/m² (i.e., the maximum deposited cisplatin dose obtained in phase I, reached over a total of more than 6 h nebulization per cycle [7]) can be delivered by means of a DPI device from a maximum of 4 capsules filled with 20 mg of powder, i.e., in less than 5 min in total per cycle. Similar results have been obtained with paclitaxel-based dry powders characterized by drug loading values of up to 75% [29].

Lung cancer patients often present highly reduced lung function due to the presence of lung tumor(s) but also to subjacent chronic lung diseases (e.g., COPD) and tobacco-smoking history. Delivering inhaled therapies, especially by means of a DPI activated and driven only by the patients’ inspiratory flow, might therefore be challenging. However, as with asthma and COPD patients, low resistance DPI should be used as they require a lower inspiratory effort to reach the airflow that allows a good aerosolization, dispersion and finally lung deposition of the powder [37]. Moreover, a positive remark is that lung cancer patients are usually already trained to use DPIs because of chronic treatments they received for several years for their pulmonary diseases (e.g., COPD).

Moreover, DPI can manage the large contamination generated by the toxic aerosol during nebulization. As DPIs are only activated and driven by the patients’ inspiratory flow for several seconds, drug concentrations found in the air and that are the consequence of drug exhalation only are negligible (i.e., 0.2% of total dose in a study with DPI tobramycin in healthy subjects [38]). Newly designed DPI devices should be developed to be adapted to chemotherapy.

‘Smart’ inhalers are inhalation devices that are able to target a specific zone in the respiratory tract. As mentioned above, lung tumor site(s) could be poorly ventilated due to the presence of a tumor mass that can negatively affect drug deposition in the lungs [6]. However, it can also positively influence aerosol deposition [30]. A decrease in the bronchi diameter, resulting from the presence of solid tumors and/or excessive production of mucus (i.e., as encountered with various histologies such as in invasive mucinous adenocarcinoma [39]), can increase air turbulence in this zone and therefore increase drug deposition by impaction [30]. These modifications could be exploited to design ‘smart’ inhalers able to increase aerosol deposition in these zones, in the tumor site(s). ‘Smart’ nebulizers are able to control critical patient’s inspiratory parameters such as inspiratory flow rate, inspiratory volume and time during the inspiration to adapt aerosol characteristics and delivery parameters. An in silico study has been made with this aim [30]. The authors demonstrated that it was possible to increase the deposited fraction of the aerosol on a tumor surface from 5–10% to 35–92% in normal vs. controlled conditions, respectively. The deposited fraction in untargeted zones decreased respectively from 20–25% to 5–15%, which could decrease local adverse effects on healthy tissues. A phase I trial has recently been initiated (ClinicalTrials.gov identifier: NCT03326752) to evaluate the compound DV 281 (i.e., a toll-like receptor 9 agonist) for the treatment of lung cancer by inhalation using a smart nebulizer, i.e., AKITA^®^ [32], to improve pulmonary delivery efficiency.

In addition to the use of smart nebulizers discussed above, nebulizer adaptations in clinical trials are discussed in Section 2.3.

It should be remarked that, although pressurized metered dose inhalers (pMDIs) are very common in the treatment of asthma and COPD, they are not suitable for chemotherapy. This is because of their inability to deliver high drug doses to the lungs (i.e., typically in the microgram range) [19].

### 3.2. Tailored Chemotherapy Formulations

#### 3.2.1. Controlled-Release DPI Formulations

**Figure 1:**
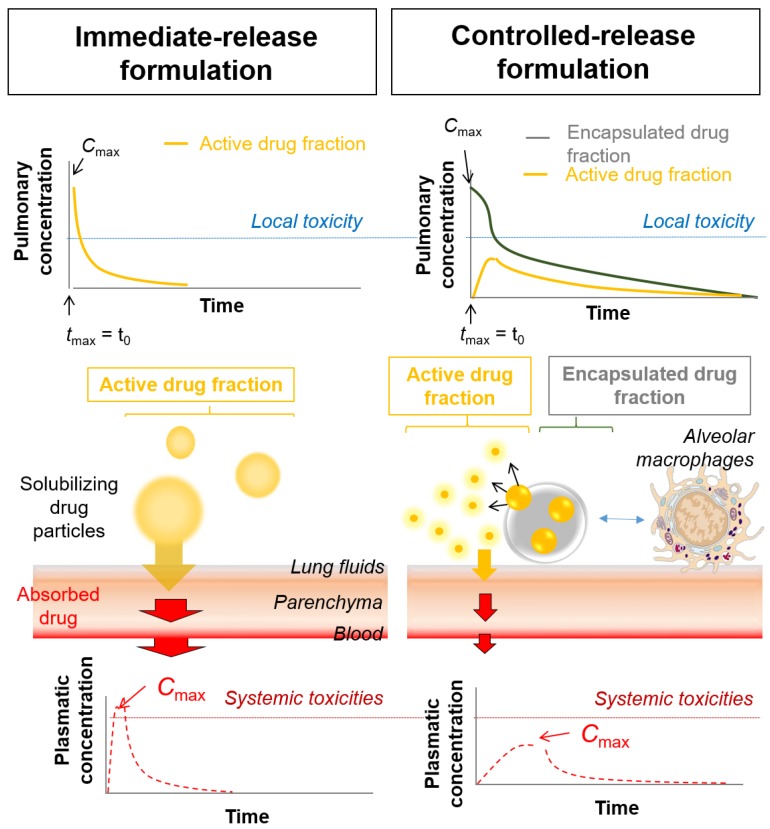
Immediate vs. controlled-release formulation for inhalation: local and systemic pharmacokinetic profiles.

Depositing high drug doses in the lungs is preferable to inhaled chemotherapy, as with DPIs. However, this might lead to high concentration peaks in lung fluids and tissues, inducing poor local tolerance. DPIs differ from conventional nebulization, for which a long administration time is needed. Conventional nebulization has led to flattened concentration peaks, possibly explaining why acceptable local adverse effects have been observed for most of the chemotherapies investigated [3,9]. This might not be the case with DPIs. Therefore, controlled-release formulations might be of interest to avoid high local active concentration peaks (i.e., corresponding to the dissolved part of the dose that has been released from a controlled-release formulation) and ensure local tolerance (Figure 1) [19,20].

Moreover, prolonging drug retention can also be profitable for inhaled chemotherapy by maintaining therapeutic concentrations in the lung fluids. However, a sustained-release profile is challenging because inhaled drugs, either in solution or as particles, are rapidly cleared from the lungs. The main elimination mechanisms include systemic absorption for solubilized and permeable drugs (i.e., in blood and/or lymphatic circulation) and clearance mechanisms for non-solubilized drugs or particles (i.e., mucociliary clearance and macrophage uptake in the upper and smaller airways, respectively) [40]. Examples of controlled-release DPI formulations that have been developed to bypass these clearance mechanisms are PEGylated solid lipid microparticles (PEG-SLMs) and large porous particles (LPPs).

Cisplatin-loaded PEG-SLM have been investigated in vivo as dry powders for inhalation [41,42]. PEG-SLM have prolonged platinum retention in the lungs to 8 h due to the controlled-released profile from the lipid matrix and the ‘stealth’ properties of the PEG coating. [42]. Moreover, PEG-SLM have increased tolerance of healthy mice to inhaled cisplatin, with a doubled cisplatin maximum tolerated dose (MTD) compared with that from a nebulized cisplatin solution and an immediate-release DPI formulation (1 vs. 0.5 mg/kg, respectively) [41]. Low inflammation and cytotoxicity directly imputable to cisplatin have been observed in broncho-alveolar lavage fluids (BALF) following a single administration (at MTD) of PEG-SLM in healthy mice [41]. The results showed significant neutrophil recruitment, a non-statistically significant (NS) decrease in total cell number, an NS increase in IL-6 concentrations and similar total protein, IL-1β and TNF-α concentrations and LDH activity, compared with non-treated control mice. PEG-SLM at 1 mg/kg allowed a similar in vivo survival rate to that from iv cisplatin at 0.5 mg/kg to be maintained in the aggressive M109 murine lung cancer model [41].

Another example of prolonged lung retention is the LPP. LPPs are designed with optimal size properties for bypassing the uptake by alveolar macrophages, i.e. typically geometric size between 5 and 50 µm, and a low density to ensure an aerodynamic diameter for deposit in the lungs. Endotracheal delivery of paclitaxel-loaded LPPs in rats maintained paclitaxel plasma concentrations (i.e., which give an indication of the paclitaxel fraction dissolved in the lungs) in the therapeutic range 4-fold longer than from the same iv dose [43]. In this study, the lung targeting efficiency of the LPPs was almost 12-fold higher than iv administration.

Selection of the proper formulation and excipients will depend on the physicochemical properties of the drug. For example, a lipid matrix (i.e., as in SLM) will be preferred for the encapsulation of hydrophobic compounds (i.e., most of the chemotherapeutic drugs) [21,44].

However, attention should be paid regarding therapeutic concentrations in tumor tissues when using controlled-release profile. Controlled-release DPI formulations allow to deliver high drug doses to the lungs within a short time period while flattening and prolonging retention of active concentration peaks in lung fluids. These low and prolonged concentration peaks are similar as the concentration peaks observed with nebulization, as in the study with nebulized carboplatin [9]. As discussed above, combining the administration routes for carboplatin (i.e., iv and pulmonary routes) in a carboplatin/docetaxel doublet significantly increased survival rates compared to the same conventional iv doublet (275 days (95% CI 249–300) vs. 211 (95% CI 185–236)), while the use of inhalation alone did not. The investigators concluded that the lower success of inhalation alone was due to the low concentrations in tumor tissues and lymph nodes obtained with the nebulizer (because of the long time needed to deliver the dose with this device) [9]. Therefore, the use of sustained-release formulations for inhalation might only be useful if combined with conventional iv perfusion.

#### 3.2.2. Nanomedicine

**Figure 2:**
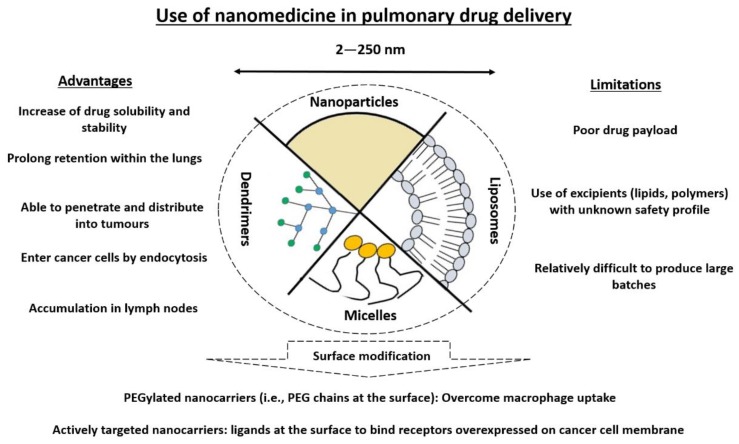
Advantages and limitations of nanomedicine in pulmonary drug delivery (adapted from [19]).

Another strategy is the use of nanomedicine, i.e., the application of nanotechnology in medicine. This refers to the use of drug carriers elaborated at nanoscale, i.e., nanocarriers, typically in the range of 100 nm [45]. The main advantage of nanomedicine in oncology is an improvement in the pharmacokinetic profile of the carried drug, with a preferential accumulation in tumors in some solid cancers [46]. This improvement is induced by passive targeting through the enhanced permeation and retention (EPR) effect or active targeting though the active pathway (e.g., receptor-mediated endocytosis) of tumor tissues or cells, respectively [47,48]. The paclitaxel-based formulation Abraxane^®^ [49] is the only nanomedicine approved by EMA and the FDA in combination with carboplatin for the first-line treatment of advanced NSCLC in adult patients who are not candidates for potentially curative surgery and/or radiation therapy (EMA Procedure No. EMEA/H/C/000778/II/0067). Nanomedicines are also considered for pulmonary drug delivery as they present many advantages in pulmonary drug delivery (Figure 2).

**Figure 3:**
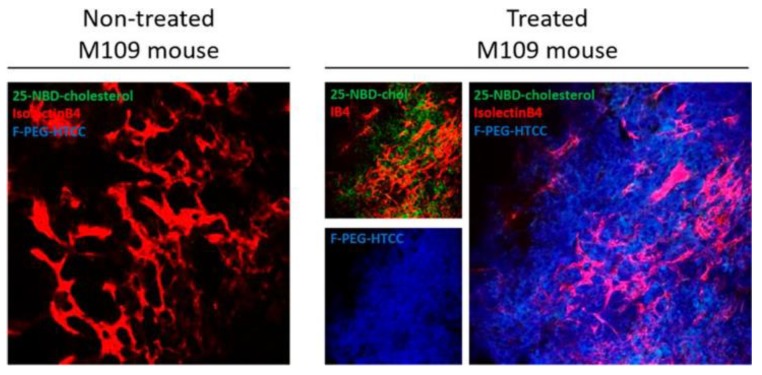
In vivo tumor distribution of coated fluorescent solid lipid nanoparticles (SLN) after administration by inhalation on the M109 model. Confocal pictures of control untreated M109 mouse lung and coated fluorescent SLN-treated mouse lung. Red: Vessels labelled with isolectinB4; green: 25-NBD-cholesterol labelling SLN; blue: Alexa Fluor 405 labelling the coating (from [37]).

Rosière et al. designed a nanocarrier loaded with paclitaxel for inhalation [33]. The nanocarriers had the particularity of presenting at their surface a folate-grafted copolymer to (i) prolong retention in the respiratory through bioadhesive properties and (ii) target the folate receptor-alpha overexpressed on plasma membrane of lung tumor cells. The nanocarrier sustained the release profile and prolonged retention of paclitaxel to 7 h within the lungs in healthy mice. The notable outcome in this study was the ability of the nanocarrier to penetrate murine M109 lung tumors in vivo (Figure 3).

**Figure 4:**
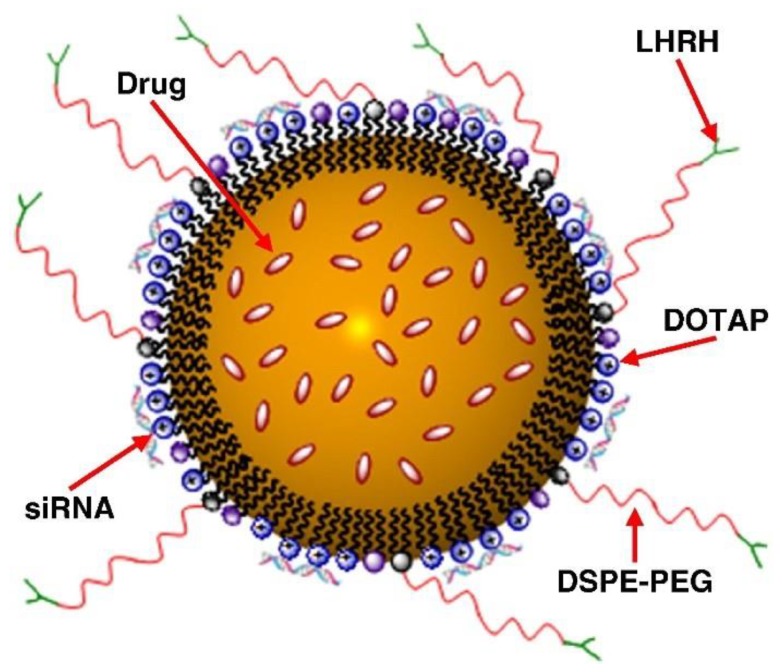
A schematic representation of a nanostructured lipid carrier (NLC)-based drug delivery system for pulmonary co-delivery of an anticancer drug, siRNA and targeting peptide (from [38]).

Taratula et al. developed a multifunctional nanocarrier that includes many components tending to improve therapeutic response (Figure 4) [34]. Paclitaxel or doxorubicin was entrapped in a positively-charged (DOTAP) lipid nanocarrier which was coated with siRNA (silencing MRP1 and BCL2, involved in pump and non-pump resistance, respectively), poly(ethylene glycol) chains (DSPE-PEG) conferring ‘stealth’ properties and a targeting moiety (an analogue of luteinizing hormone-releasing hormone (LHRH)) to target lung cancer cells. Targeting was demonstrated in vivo in an orthotopic lung tumor mice model (human A549 adenocarcinoma tumors) with (i) minor distribution of the nanocarrier in untargeted organs (compared with iv) and (ii) preferential delivery in lung cancer cells and leaving healthy lung tissues intact [34]. Antitumor activity was also enhanced compared with iv, with a ~40-fold decrease in tumor volume and allowing complete regression in 50% of mice [34]. 

However, a central limitation of nanomedicine pulmonary drug delivery is the poor drug loading capacity of the nanocarrier (usually in the range 1–10%) (Figure 2). In addition to potentially not being able to deliver therapeutic doses, a poor payload requires the administration of high doses of excipients that are responsible for poor local tolerance [50]. Toxicity is one of the main concern in nanomedicine for pulmonary drug delivery [50].

## 4. The Position of Inhaled Chemotherapy in Patient Care: Potential Chemotherapy and Future Indications

Increasing the therapeutic ratio of chemotherapy would be the main advantage using the pulmonary route. The clinical data and recent preclinical development discussed above indicate that inhaled chemotherapy now seems to be a realistic approach to include in further clinical development. Selection of the proper subpopulations of patients is primordial. This selection must be made according to tumor localization and size and therefore the clinical stage and cancer histology as well as the patient’s pulmonary function and subjacent lung diseases [15]. Three potential future indications have been suggested in the literature. Inhaled chemotherapy, mostly in association with other treatment approaches, is realistic in advanced stage lung cancers, and could also be useful as a neoadjuvant and adjuvant therapy for localized and locoregional stage lung cancers and in chemoprevention of pulmonary metastases [9,10].

Firstly, in advanced stage lung cancers, inhaled chemotherapy would be combined with systemic therapies. This strategy would combine a chemotherapy with significant systemic toxicities (e.g., cisplatin, carboplatin, paclitaxel), delivered by inhalation or by both inhalation and iv, with other compounds with relatively lower systemic side effects (e.g., gemcitabine, pemetrexed). Very promising clinical results have been obtained with this strategy by administering carboplatin through the iv and pulmonary routes [9].

Secondly, as a neoadjuvant and adjuvant therapy, inhaled chemotherapy could be used in combination with other neoadjuvant or adjuvant therapies (e.g., radiotherapy, systemic chemotherapy) to limit loco-regional relapses. These relapses result from microscopic non-resected tumor tissues. This strategy seemed promising in the resection of pulmonary metastases of osteosarcoma (tumors ≤ 2 cm resected), with a complete remission observed in a patient [11].

Thirdly, although chemoprevention of pulmonary metastases has not been investigated, some authors have suggested this strategy as potential indication of inhaled chemotherapy [10]. Some cancers form metastases preferentially in the lungs, e.g., more than 75% of osteosarcoma [11].

Moreover, as mentioned above, the type of lung cancer according to histology should also be of interest when selecting patients for inhaled chemotherapy. Adenocarcinoma with lepidic pattern, not only as predominant pattern but also as secondary pattern, should be a good candidate. According to the latest WHO classification of lung tumors, four histologic types can present a lepidic pattern [39]. Two of them, i.e., adenocarcinoma in situ (AIS) and minimally invasive adenocarcinoma (MIA) are characterized, if treated properly, by 100% disease-free survival and are therefore relatively less subject, at least at this stage of clinical development, to optimization of treatments [39]. However, lepidic adenocarcinoma and particularly mucinous invasive adenocarcinoma are characterized by intermediate to bad prognosis, respectively [39]. In this type of lung tumor, inhaled chemotherapy could be, as suggested above for advanced stage lung cancers, a platinum salt in association with iv pemetrexed or taxane (i.e., docetaxel, paclitaxel or nab-paclitaxel). Pemetrexed or taxanes could also be envisaged for inhalation to reduce the systemic toxicities, as observed with paclitaxel in preclinical studies in rodents [51,52] and dogs [35]. In this strategy, inhaled paclitaxel would be combined with iv platinum salt and/or be used in maintenance.

Finally, another approach might be to combine inhaled chemotherapy with immunotherapy. Recent approval of immune checkpoint inhibitors (e.g., pembrolizumab, nivolumab, atezolizumab) in the therapeutic arsenal as first-line and/or second-line therapy has brought new perspectives in the care of NSCLC patients. However, substantial patient populations present tumors that remain insensitive to these inhibitors [53]. Nevertheless, longer overall survivals have been observed in patients expressing low levels of PD-L1 compared with chemotherapy [54,55,56] which has been recently demonstrated to modulate the tumor environment and play a positive role in the immunogenic response. The rationale for the combination of immunotherapy with conventional chemotherapy has been recently reviewed [57,58]. Inhaled chemotherapy, with its potentially better safety profile, might be preferred over systemic chemotherapy as an activator to immunotherapy. Clinical studies are now required to demonstrate this potentially promising application.

## 5. Conclusions

Use of the pulmonary route is a promising way to decrease the severe systemic toxicities associated with chemotherapy. Inhaled chemotherapy has been proved to be feasible and safe in phase I, Ib/IIa and II clinical trials. Inhalation allows the administration of high drug doses directly to lung tumors without prior distribution in the organism. The severe systemic toxicities are consequently reduced. However, the lack of improvement in the benefit/risk ratio, compared with conventional chemotherapy, in addition to higher financial costs (due to specific hospital facilities and the required inhalation procedure to limit environmental contamination) mean that inhalation has been underexploited in lung tumor therapy. Nevertheless, a changing trend has been observed in the past decade with the introduction of new pharmaceutical technologies such as particle engineering in DPI formulations and nanomedicine.

It is obvious that inhaled chemotherapy should be adapted to specific subpopulations of patients only and be used in well-defined clinical cases. To this end, the lepidic pattern, as the predominant or secondary pattern, seems to be a good histological type to start with. Inhaled chemotherapy could be proposed, based on clinical and preclinical data, mostly in association with other treatment modalities such as immunotherapy, in advanced cancers, or as a neoadjuvant or adjuvant therapy in more localized stages and/or in chemoprevention of lung metastases. New clinical trials are now needed to demonstrate the potential of this new therapeutic modality.

## Figures and Tables

**Table 1:** Promising technologies in relation to the challenges they take up.

	High Dose Deposition in the Lungs	Reasonable Time of Administration	Prolonged Pulmonary Residence	Potentially Safe Pulmonary Profile	Negligible Environmental Contamination	Ref.
Dry powder inhalers (DPIs)	++	++	~	~	++	[28,29]
Smart inhalers/targeted deposition	++	+	~	+	+	[30,31,32]
Controlled-release DPI formulations	+	++	++	+	++	[29,33,34,35]
Nanomedicine	~	~	++	++	~	[22,33,34]

++ highly promising; +, promising; ~subject to optimization (e.g., combination between technologies, future development).
